# Distinct roles of long/short fimbriae and gingipains in homotypic biofilm development by *Porphyromonas gingivalis*

**DOI:** 10.1186/1471-2180-9-105

**Published:** 2009-05-26

**Authors:** Masae Kuboniwa, Atsuo Amano, Ei Hashino, Yumiko Yamamoto, Hiroaki Inaba, Nobushiro Hamada, Koji Nakayama, Gena D Tribble, Richard J Lamont, Satoshi Shizukuishi

**Affiliations:** 1 Department of Preventive Dentistry, Osaka University Graduate School of Dentistry, Suita-Osaka, Japan; 2 Department of Oral Frontier Biology, Osaka University Graduate School of Dentistry, Suita-Osaka, Japan; 3 Department of Oral Microbiology, Kanagawa Dental College, Yokosuka-Kanagawa, Japan; 4 Department of Developmental and Reconstructive Medicine, Nagasaki University Graduate School of Biomedical Sciences, Nagasaki, Japan; 5 Department of Periodontics, The University of Texas Health Science Center at Houston, Houston, TX, USA; 6 Department of Oral Biology, University of Florida College of Dentistry, Gainesville, FL, USA

## Abstract

**Background:**

*Porphyromonas gingivalis*, a periodontal pathogen, expresses a number of virulence factors, including long (FimA) and short (Mfa) fimbriae as well as gingipains comprised of arginine-specific (Rgp) and lysine-specific (Kgp) cysteine proteinases. The aim of this study was to examine the roles of these components in homotypic biofilm development by *P. gingivalis*, as well as in accumulation of exopolysaccharide in biofilms.

**Results:**

Biofilms were formed on saliva-coated glass surfaces in PBS or diluted trypticase soy broth (dTSB). Microscopic observation showed that the wild type strain formed biofilms with a dense basal monolayer and dispersed microcolonies in both PBS and dTSB. A FimA deficient mutant formed patchy and small microcolonies in PBS, but the organisms proliferated and formed a cohesive biofilm with dense exopolysaccharides in dTSB. A Mfa mutant developed tall and large microcolonies in PBS as well as dTSB. A Kgp mutant formed markedly thick biofilms filled with large clumped colonies under both conditions. A RgpA/B double mutant developed channel-like biofilms with fibrillar and tall microcolonies in PBS. When this mutant was studied in dTSB, there was an increase in the number of peaks and the morphology changed to taller and loosely packed biofilms. In addition, deletion of FimA reduced the autoaggregation efficiency, whereas autoaggregation was significantly increased in the Kgp and Mfa mutants, with a clear association with alteration of biofilm structures under the non-proliferation condition. In contrast, this association was not observed in the Rgp-null mutants.

**Conclusion:**

These results suggested that the FimA fimbriae promote initial biofilm formation but exert a restraining regulation on biofilm maturation, whereas Mfa and Kgp have suppressive and regulatory roles during biofilm development. Rgp controlled microcolony morphology and biovolume. Collectively, these molecules seem to act coordinately to regulate the development of mature *P. gingivalis *biofilms.

## Background

*Porphyromonas gingivalis *has been shown to be a major etiologic agent of destructive adult periodontitis, with a significant lifestyle component harbored within the complex multi-species biofilm (dental plaque) that develops along the gingival margins [1]. The bacterium expresses a number of potential virulence factors, such as long (major) and short (minor) fimbriae, lipopolysaccharides (LPS), and proteases [2]. Among these factors, a unique class of cysteine proteinases, termed gingipains, composed of arginine-specific [Arg-gingipain A and B, (RgpA and RgpB, respectively)] and lysine-specific (Kgp) proteases, are implicated in a wide range of both pathological and physiological processes [3]. Proteases can be post-translationally processed for retention on the cell surface or secretion into the extracellular milieu. Rgp enzymes are glycosylated, with their carbohydrate domain containing phosphorylated branched mannans that can contribute to the anchoring of Rgp on bacterial outer membrane [4]. In addition, this phosphorylated branched mannan constitutes an exopolysaccharide that is distinguishable from both LPS and the serotypeable capsule polysaccharides of *P. gingivalis *[4].

The cell-associated gingipains comprise the majority (~80%) of Rgp and Kgp activities, and are reported to be definitive virulence factors that degrade various host proteins, leading to impaired cellular integrity and function [5]. In addition, gingipains can mediate bacterial interactions with host components [6]. Recent findings indicate that gingipains are also involved in biofilm development. Polyphenolic inhibitors of gingipains can prevent not only homotypic (monospecies) biofilm formation by *P. gingivalis *[7], but also synergistic biofilm formation with *Fusobacterium nucleatum *[8]. In addition, an RgpB-deficient mutant of *P. gingivalis *lost the ability to form synergistic biofilms with *Treponema denticola *[9]. A low molecular weight tyrosine phosphatase, Ltp1, was found to be involved in biofilm formation via suppression of exopolysaccharide production and *luxS *expression, as well as dephosphorylation of gingipains [10]. Thus, gingipains and gingipain regulation may be related to exopolysaccharide accumulation. However, the exact role of gingipains in biofilm development remains to be elucidated.

Two distinct fimbria types, long and short fimbriae, are present on the surface of *P*. *gingivalis *cells [11]. Long fimbriae impact the host immune response by inducing human peripheral macrophages and neutrophils to overproduce several proinflammatory cytokines such as interleukin-1 (IL-l), IL-6, and tumor necrosis factor alpha, through coordinated interactions with pattern-recognition receptors [12]. Long fimbriae were also reported to induce cross-talk between CXC chemokine receptor 4 and Toll-like receptor 2 in human monocytes and thus undermine host defense [13]. Furthermore, long fimbriae are prominent adhesins that mediate colonization in periodontal tissues and invasion of host cells as well as dysregulation of host cell cycle, which assists *P. gingivalis *in its persistence in periodontal tissues [14,15]. While, the role of short fimbriae in virulence is less well understood, they are necessary for the development of synergistic biofilms between *P*. *gingivalis *and *Streptococcus gordonii *via a specific interaction with the streptococcal SspB protein [16]. Recently, these two distinct types of fimbriae were reported to function cooperatively in the development of homotypic biofilms of *P. gingivalis *[17]. It was proposed that the long fimbriae were responsible for bacterial attachment to the substrate as well as initiation of colonization, whereas short fimbriae were involved in the formation of microcolonies and biofilm maturation. In that study, it was also shown that short fimbriae promoted bacterial autoaggregation, which was suppressed by the long fimbriae. In contrast, another study showed opposite results, as deletion of short fimbriae enhanced autoaggregation and negligible autoaggregation occurred in the long fimbria mutants tested [18]. Thus, the contextual roles of these fimbria types in biofilm development are unclear, and further study is necessary.

In the present study, we examined the roles of long and short fimbriae as well as Arg-and Lys-gingipains in homotypic biofilm formation by *P. gingivalis *using a series of deletion mutants of strain ATCC33277.

## Results

### Microstructure of biofilms under nonproliferation condition

**Figure 1:**
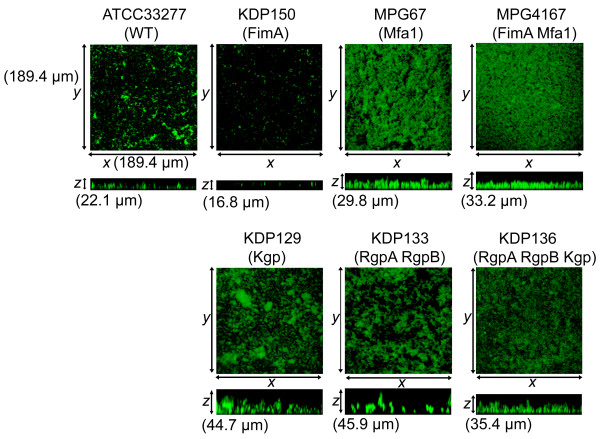
**Homotypic biofilm formation by *P. gingivalis *wild-type strain and mutants in PBS**. *P. gingivalis *strains were stained with CFSE (green) and incubated in PBS for 24 hours. After washing, the biofilms that developed on the coverglass were observed with a CLSM equipped with a 40× objective. Optical sections were obtained along the *z *axis at 0.7-μm intervals, and images of the *x*-*y *and *x*-*z *planes were reconstructed with an imaging software as described in the text. Upper panels indicate *z *stacks of the *x-y *sections. Lower panels are *x-z *sections. *P. gingivalis *strains used in this assay are listed in Table 4. The experiment was repeated independently three times with each strain in triplicate. Representative images are shown.

First, we evaluated the roles of long/short fimbriae and gingipains in initial attachment and organization of biofilms which is a crucial event in the early phase of biofilm formation [19]. When cultured in TSB as free-living cells, wild type and all mutant strains showed the similar growth rates, as reported in previous study [20]. In contrast, when incubated in PBS for 24 h, wild type and mutants lacking long and/or short fimbriae formed distinct biofilms (Figure 1 and Table 1). Wild type strain 33277 formed biofilms with a dense basal monolayer and dispersed microcolonies. Compared with the wild type, the long fimbria mutant KDP150 formed patchy and sparser biofilms with a significantly greater distance between fewer peaks, although mean peak height was almost the same as that of the wild type strain. In contrast, the short fimbria mutant MPG67 developed cluster and channel-like biofilms consisting of significantly taller microcolonies compared to the wild type. Similar to MPG67, the mutant (MPG4167) lacking both types of fimbriae also formed thick biofilms with significantly taller microcolonies than the wild type. Viability of the cells in biofilms of each strain was tested by colony count and confirmed at 24 h (data not shown). These results suggest that the long fimbriae are involved in initial attachment and organization of biofilms by *P. gingivalis*, whereas the short fimbriae have a suppressive regulatory role for these steps.

**Table 1:** Features of biofilms formed by *P. gingivalis *wild-type strain and mutants in PBS

	Peak parameters^a)^		
	
Strain	Number of peaks	Mean distance between peaks (μm)	Mean peak height (μm)
ATCC33277 (wild type)	28.5 ± 3.3	3.0 ± 0.2	2.8 ± 0.4
KDP150 (Δ*fimA*)	14.7 ± 2.4**	5.4 ± 1.0**	2.7 ± 0.8
MPG67 (Δ*mfa1*)	29.3 ± 2.0	3.6 ± 0.2	16.6 ± 0.8**
MPG4167 (Δ*fimA*Δ*mfa1*)	30.5 ± 1.9	3.1 ± 0.2	12.7 ± 0.5**
KDP129 (Δ*kgp*)	25.5 ± 2.1	3.6 ± 0.3	12.7 ± 1.3**
KDP133 (Δ*rgpA*Δ*rgpB*)	13.0 ± 2.6**	8.4 ± 1.3**	23.2 ± 2.8**
KDP136 (Δ*rgpA*Δ*rgpB*Δ*kgp*)	30.5 ± 2.4	3.2 ± 0.2	12.7 ± 0.7**

^a) ^Number of peaks was evaluated in an area sized 90 (*x *axis) × 2 (*y *axis) μm. The mean ± SE of 10 areas was shown.

**p < 0.01 in comparison with the wild type using a Scheffe test.

The involvement of gingipains in biofilm formation was evaluated using a set of *P. gingivalis *mutants lacking Kgp (KDP129), RgpA/B (KDP133), or both Kgp and RgpA/B (KDP136). These mutants lacked the proteolytic domains as well as the adhesion domains of gingipains [5]. In addition, both Rgp mutants (KDP133 and KDP136) lacked bacterial cell-surface structural components such as long and short fimbriae and hemagglutinins which are processed by Rgp [21-23]. The Kgp mutant KDP129 formed markedly thick biofilms containing large accumulations of which the mean height was significantly taller than the wild type (Figure 1 and Table 1). In addition, the efficiency of autoaggregation in KDP129 was significantly increased (Table 2). These results suggest that Kgp plays a negative role in biofilm development via suppressing autoaggregation and/or regulating dispersion, de-concentration, and/or detachment of microcolonies. The RgpA/B mutant KDP133 formed channel-like biofilms with fibrillar microcolonies (Figure 1), which featured significantly fewer peaks and longer distances between peaks, but increased height, as compared to those of the wild type and Kgp mutant (Table 1). Although the features of KDP133 were likely attributable to the loss of multiple factors on the bacterial surface, Rgp itself might be a bifunctional mediator promoting peak formation and shearing the fibrillar microcolonies of biofilms. Interestingly, the biofilms formed by the gingipain null mutant (KDP136) showed different features from both the Kgp (KDP129) and Rgp (KDP133) mutants. Although the three mutants, KDP136, KDP133 and MPG4167, resemble each other in terms of lack of expression of both types of fimbriae, their microstructures were divergent (Figure 1). These findings suggested that biofilm formation was affected not only by the post-translational regulation of the expression of cell surface components by Rgp, but also by uncharacterized steps that were not altered by Rgp. Loss of all gingipain activities might result in downstream events which did not happen in KDP129 and KDP133.

**Table 2:** Autoaggregation of *P. gingivalis *wild-type strain and mutants

Strain	Autoaggregation index^a)^ (-dA/min)
ATCC33277 (wild type)	17.73 ± 1.67
KDP150 (Δ*fimA*)	0.54 ± 3.94**
MPG67 (Δ*mfa1*)	36.12 ± 2.40**
MPG4167 (Δ*fimA*Δ*mfa1*)	33.87 ± 2.77**
KDP129 (Δ*kgp*)	35.62 ± 2.52**
KDP133 (Δ*rgpA*Δ*rgpB*)	15.04 ± 2.68
KDP136 (Δ*rgpA*Δ*rgpB*Δ*kgp*)	0.29 ± 3.22**

^a) ^dA/min was automatically calculated by subtraction of At, the absorbance at time t min, from At+, at time (t + 1) min during incubation. The maximum value of – dA/min in a curve was used as the autoaggregation index. The data represent the mean ± SE of three separate experiments with each strain in duplicate.

**p < 0.01 in comparison with the wild type using a Scheffe test.

### Quantitative analysis of biofilms in PBS

**Figure 2:**
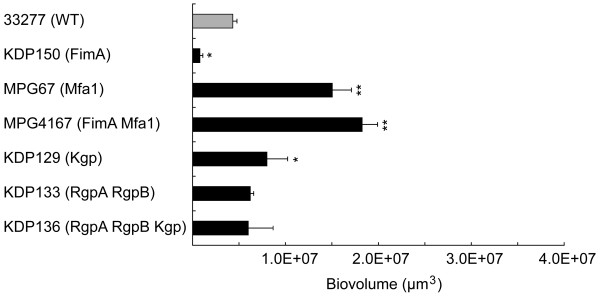
**Quantification of homotypic biofilms formed by *P. gingivalis *wild-type strain and mutants in PBS**. Biofilms were formed as described in Figure 1, and 10 fields per a sample were randomly recorded and quantified with a CLSM. *Z *stacks of the *x-y *sections were converted to composite images to quantify each biovolume as described in the text. Standard error bars are shown. Statistical analysis was performed using a Scheffe test. *p < 0.05 and **p < 0.01 in comparison to the wild-type strain. *P. gingivalis *strains used in this assay are listed in Table 4.

The biovolume of the biofilms was also altered by deletion of various bacterial factors (Figure 2). The deletion of long fimbriae significantly reduced the biovolume, whereas the mutant without short fimbriae developed extensive biofilms. The deletion of Kgp also increased the biovolume, whereas no significant change was observed in the Rgp mutants. These results support the above suggested roles; i.e., long fimbriae are a facilitator, short fimbriae and Kpg are suppressors, whereas Rgp has dual functions, promoting peak formation and shearing the fibrillar microcolonies, in the initial phase of biofilm formation by *P. gingivalis*.

### Microstructure under proliferation condition

**Figure 3:**
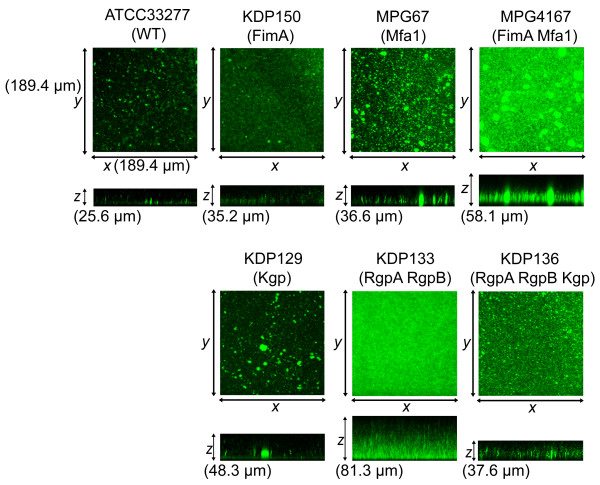
**Homotypic biofilm formation by *P. gingivalis *wild-type strain and mutants in dTSB**. *P. gingivalis *strains were stained with CFSE (green) and incubated in dTSB for 24 hours. After washing, the biofilms that developed on the coverglasses were observed with a CLSM equipped with a 40× objective. Optical sections were obtained along the *z *axis at 0.7-μm intervals, and images of the *x*-*y *and *x*-*z *planes were reconstructed with imaging software, as described in the text. Upper panels indicate *z *stacks of the *x-y *sections. Lower panels show *x-z *sections. The experiment was repeated independently three times with each strain in triplicate. Representative images are shown.

Next, the roles of the fimbriae and gingipains were examined in the early maturation phase of biofilms, which is associated with an increase in biovolume mainly due to cell division and exopolysaccharide accumulation. Biofilm development was induced by culture in nutrient medium. Figure 3 shows various features of biofilms of the mutants incubated in dTSB for 24 hours. The wild type strain formed biofilms with a dense basal monolayer with dispersed microcolonies, similar to the PBS condition, but with more and taller peaks (Table 3). The long fimbria mutant KDP150 formed biofilms with a thicker monolayer and with a greater number of the fine, taller peaks compared to wild type, (Figure 3 and Table 3). Those features suggested that long fimbriae have a role in suppression of the development of an thickened basal layer, but trigger protruding peak formation in early maturation phase. The short fimbria mutant MPG67 formed significantly clustered biofilms consisted of tall and wide microcolonies, suggesting that short fimbriae negatively control the morphology of microcolonies, as mentioned above. The mutant lacking both types of fimbriae (MPG4167) also formed markedly thick and dense biofilms containing various size of microcolonies, suggesting that both types of fimbriae negatively regulate biofilm formation in early maturation phase. The Kgp mutant KDP129 formed large microcolonies which were well dispersed, whereas the Rgp mutant KDP133 made the most thick biofilms with the tallest acicular microcolonies (Figure 3 and Table 3). These findings suggested that Kgp suppresses microcolony expansion, whereas Rgp mediates transverse enlargement and restrains the longitudinal extension. As with the result in PBS, biofilms with the gingipain null mutant KDP136 showed different features from both KDP129 and KDP133.

**Table 3:** Features of biofilms formed by *P. gingivalis *wild-type strain and mutants in dTSB

	Peak parameters^a)^		
	
Strain	Number of peaks	Mean distance between peaks (μm)	Mean peak height (μm)
ATCC33277 (wild type)	45.5 ± 3.5	2.0 ± 0.9	6.9 ± 1.4
KDP150 (Δ*fimA*)	52.5 ± 3.5*	1.7 ± 0.7*	23.7 ± 5.6**
MPG67 (Δ*mfa1*)	35.8 ± 3.6**	2.7 ± 1.6**	20.9 ± 4.4**
MPG4167 (Δ*fimA*Δ*mfa1*)	32.3 ± 3.8**	3.0 ± 1.6**	20.5 ± 4.3**
KDP129 (Δ*kgp*)	39.8 ± 3.2	2.2 ± 1.2	19.6 ± 5.4**
KDP133 (Δ*rgpA*Δ*rgpB*)	41.0 ± 5.7	2.2 ± 1.0	45.9 ± 4.5**
KDP136 (Δ*rgpA*Δ*rgpB*Δ*kgp*)	43.0 ± 1.4	2.1 ± 0.8	22.2 ± 2.4**

^a)^Number of peaks was evaluated in an area sized 90 (x axis) × 2 (y axis) μm. The mean ± SE of 10 areas was shown.

*p < 0.05 and **p < 0.01 in comparison with the wild type using a Scheffe test.

### Quantitative analysis of biofilms in dTSB

**Figure 4:**
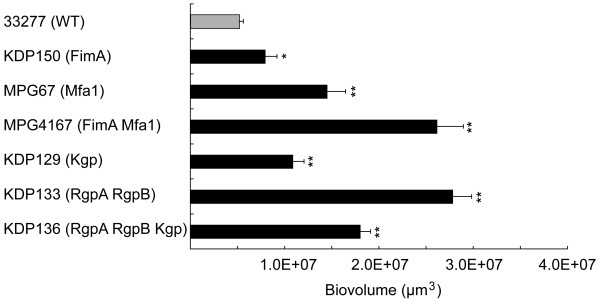
**Quantification of homotypic biofilms formed by *P. gingivalis *wild-type strain and mutants in dTSB**. Biofilms were formed as described in the legend to Figure 3, and 10 fields per a sample were randomly recorded and quantified, similar to the method described in the legend to Figure 2. Statistical analysis was performed with a Scheffe test. *p < 0.05 and **p < 0.01 in comparison to the wild-type strain.

In the early maturation phase, the biovolumes of the biofilms were significantly increased in all of tested mutants as compared to the wild type (Figure 4). Deletion of long fimbriae resulted in the opposite tendency from the initial attachment phase, suggesting that this molecule has distinct roles under the different phases.

### Exopolysaccharide production under proliferation conditions

**Figure 5:**
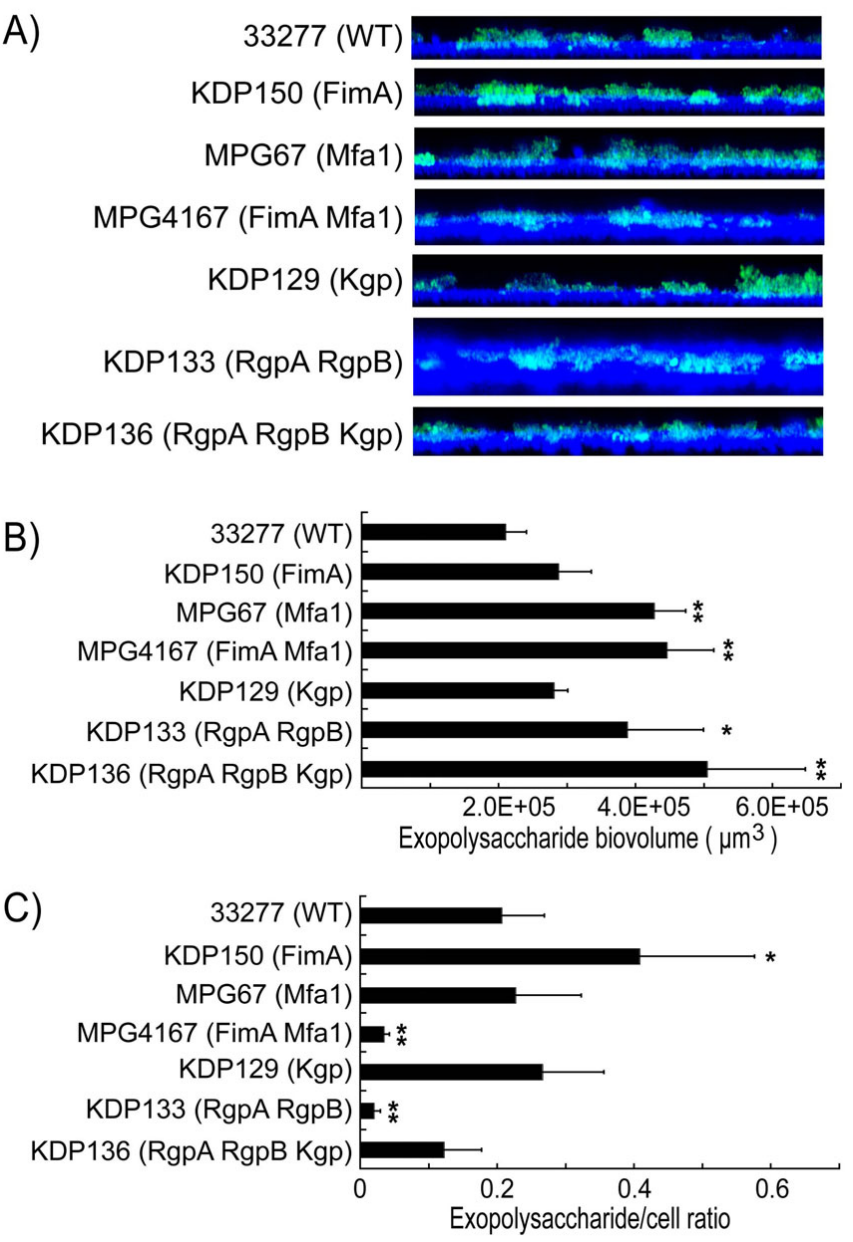
**Exopolysaccharide production by *P. gingivalis *wild-type strain and mutants in dTSB**. A) Visualization of exopolysaccharide production in biofilms formed by *P. gingivalis *strains after staining with FITC-labelled concanavalin A and wheat germ agglutinin (green). Bacteria were stained with DAPI (blue). Fluorescent images were obtained using a CLSM. The *z *stack of the *x-y *sections was converted to composite images with the "Volume" function using Imaris software, after which a *y *stack of the *x-z *sections was created and is presented here. B) Fluorescent images were quantified using Imaris software and average of total exopolysaccharide biovolume per field was calculated. C) Exopolysaccharide levels are expressed as the ratio of exopolysaccharide/cells (FITC/DAPI) fluorescence. The experiment was repeated independently three times. Data are presented as averages of 8 fields per sample with standard errors of the means. Statistical analysis was performed using a Scheffe test. *p < 0.05 and **p < 0.01 in comparison to the wild-type strain.

As extracellular polysaccharide is important for the development of biofilm communities, we examined the influences of fimbriae and gingipains on the accumulation of exopolysaccharide in *P. gingivalis *biofilms. To visualize and quantify exopolysaccharide accumulation in biofilms under the proliferation condition, 4',6-diamino-2-phenylindole (DAPI)-labeled *P. gingivalis *cells and fluorescein isothiocyanate (FITC)-labeled exopolysaccharide were examined by confocal microscopy with digitally reconstructed image analysis. In all of the tested strains, DAPI-labeled cells exhibited the same microstructures of biofilms composed of 5-(and-6)-carboxyfluorescein succinimidyl ester (CFSE)-labeled cells, as shown in Figure 3, thus validating the use of these live-staining methods (data not shown). Exopolysaccharide visualization enabled us to assess the accumulation pattern (Figure 5A) and exopolysaccharide biovolume per base area (Figure 5B). Furthermore, the exopolysaccharide production was normalized to the levels of DAPI-labeled *P. gingivalis *cells in the biofilms and expressed as the exopolysaccharide/cell ratio (Figure 5C). Interestingly, a unique pattern of exopolysaccharide accumulation was observed in the Rgp mutant KDP133 in vertical sections (*x-z *plane) of biofilms (Figure 5A). In contrast to the other strains, exopolysaccharide accumulated in the middle layer, and the biofilm surface was not covered with exopolysaccharide. It was also notable that the long fimbria mutant KDP150 developed a biofilm enriched with exopolysaccharide (Figure 5A), reflecting a significantly higher exopolysaccharide/cell ratio (Figure 5C). The gingipain null mutant KDP136 produced the most abundant exopolysaccharide per unit base area (Figure 5B). The minor fimbria mutant MPG67, long/short fimbriae mutant MPG4167 and Rgp mutant KDP133 also accumulated significantly larger amounts of exopolysaccharide than wild type; however, exopolysaccharide/cell ratio in KDP133 and MPG4167 was significantly lower than wild type because biofilms of these strains consisted of larger numbers of cells (Figure 5C).

### Autoaggregation

Bacterial autoaggregation has been reported to play an important role in initial biofilm formation [24], thus the autoaggregation efficiencies of the mutants were assessed (Table 2). Deletion of long fimbriae significantly reduced the autoaggregation efficiency, which agreed with the previous report that long fimbriae were required for autoaggregation [25]. The efficiency of autoaggregation was significantly increased in the Kgp mutant KDP129, short fimbria mutant MPG67 and long/short fimbriae deficient mutant MPG4167, suggesting that Kgp and short fimbriae act to suppress autoaggregation. Contrary to our prediction, the gingipain null mutant KDP136 and Rgp mutant KDP133 showed different tendencies of autoaggregation from MPG4167, although all of these strains were considered to be long/short fimbriae deficient mutants. Thus, not only fimbrial expression but also other factors, modified by gingipains, seem to be involved in autoaggregation. In addition, it was found that autoaggregation and biofilm parameters such as biovolume, number of peaks and peak height were not significantly correlated in every strain (Figure 2, Figure 4, Table 1 and Table 3). This result suggests that autoaggregation is not the sole determinant of alteration in structure of *P. gingivalis *biofilms.

### Tenacity of biofilms

**Figure 6:**
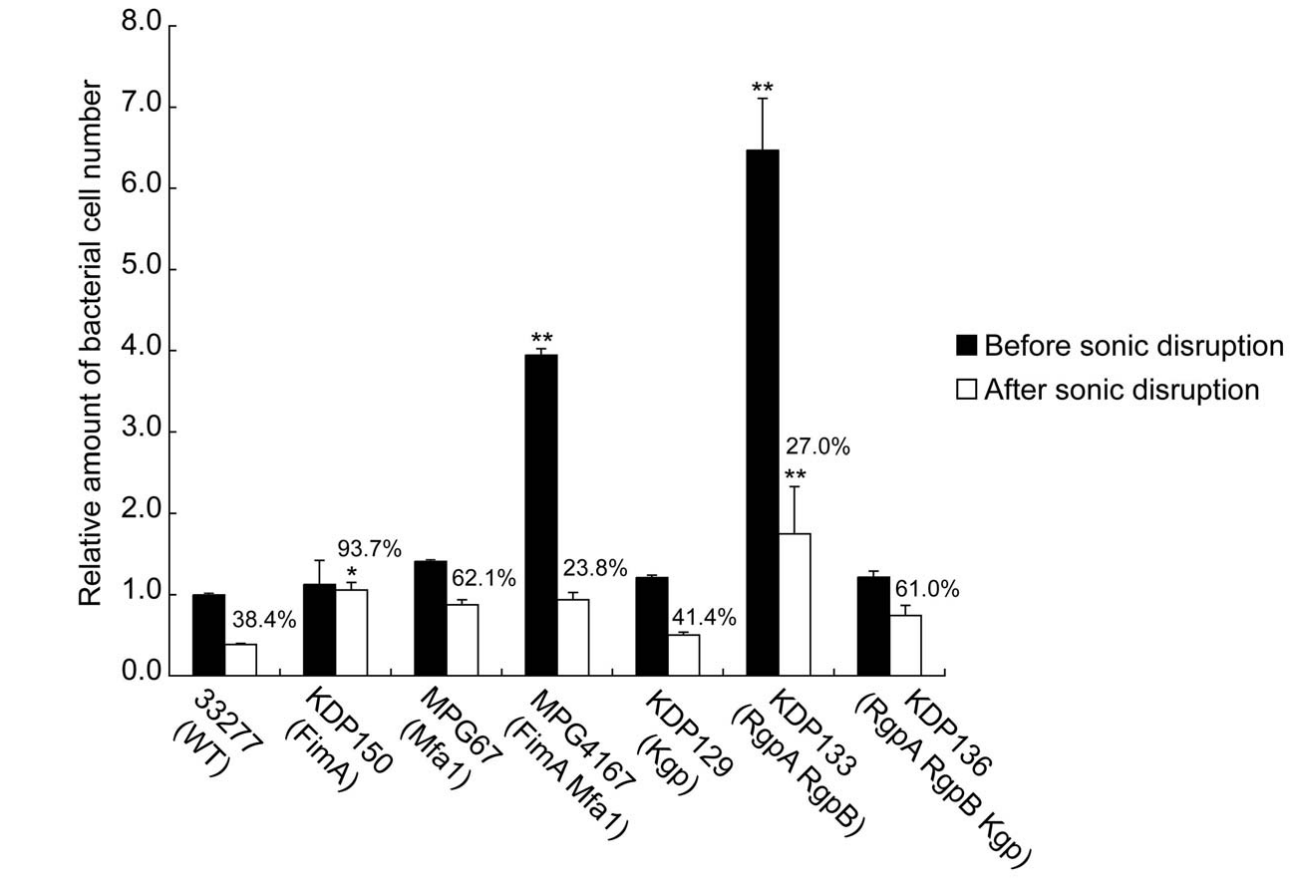
**Tenacity of biofilms formed by *P. gingivalis *wild tstrain and mutants**. Standardized cultures of *P. gingivalis *were inoculated into dTSB in saliva-coated 12-well polystyrene plate and incubated in a static manner at 37°C for 60 hours, with the resulting biofilms sonicated for 1 second. Immediately after sonication, supernatants containing floating cells were removed by aspiration and the biofilm remains were gently washed with PBS. *P. gingivalis *genomic DNA was isolated from the biofilms and the numbers of *P. gingivalis *cells were determined using real-time PCR. Relative amounts of bacterial cell numbers were calculated based on the number of wild-type cells without sonication considered to be 1.0. Percentages shown indicate the amount of remaining biofilm after sonic disruption. The experiment was repeated independently three times with each strain in duplicate. Standard error bars are shown. Statistical analysis was performed using a Scheffe test. *p < 0.05 and **p < 0.01 in comparison to the wild-type strain.

To analyze the influence of the molecules under investigation on vulnerability of biofilms, the physical strength of the biofilms against brief ultrasonication was compared (Figure 6). Consistent with the results of image analysis described in Figure 4 and Figure 5A, the long/short fimbriae mutant MPG4167 and Rgp mutant KDP133 formed expansive biofilms with large numbers of cells in dTSB, however, their strength was found to be very fragile compared to the other strains, suggesting that these biofilms consisted of loosely connected microcolonies. In contrast, the biofilms of the long fimbria mutant KDP150 were resistant to sonic disruption, suggesting that long fimbriae are initial mediator of biofilm formation but are not required to maintain resistance against environmental shear force.

Collectively, these results suggest that long fimbriae are required for initial formation of biofilms by *P. gingivalis*, but suppress the development of an exopolysaccharide-enriched basal layer that is related to the adhesive property of biofilms. In contrast, short fimbriae and Kgp may have suppressive and regulatory roles for biofilm formation, with control over morphology of microcolonies, whereas Rgp mediates microcolony formation and restrains the biovolume. In addition, other factors beside fimbriae and gingipains are likely involved in homotypic biofilm formation by *P. gingivalis*.

## Discussion

Dental plaque, a precursor for periodontal disease, is also a well studied model of bacterial biofilms in general [26,27]. Developing biofilm communities in the oral cavity are fundamental for the persistence of organisms such as *P. gingivalis *and continual exposure of the host to *P. gingivalis *can result in a dysfunctional immune response [28]. Biofilm maturation proceeds through a series of developmental steps involving the attachment of cells to, and growth on, a surface, followed by detachment and dissemination to a new site to start the cycle again [29,30]. It is likely that much of biofilm-specific physiology is devoted to dynamic changes that both stimulate an increase in biovolume and limit or stabilize accumulation according to environmental constraints. Therefore, multiple bacterial factors are thought to be required to regulate appropriate biofilm structure.

In the present study, the roles of long/short fimbriae and gingipains on the initiation and development of biofilms formed by *P. gingivalis *were examined. Interestingly, those molecules were found to play distinct roles in the above-mentioned dynamic changes that stimulate, limit or stabilize the biofilm formation. Long fimbriae were shown to be initial positive mediators of biofilm formation, however, these appendages also functioned to decrease the adhesive property of biofilms via repressing exopolysaccharide accumulation in basal layer. In addition, short fimbriae as well as Kgp were found to be negative regulators of microcolony formation and of biovolume. Rgp seems to play a bifunctional role in coordinating the integrity of the biofilm through mediating microcolony formation and restraining the biovolume. Our results indicate that all of these interactions are likely to be coordinately essential for the initiation and development of appropriately structured biofilms. To our knowledge, this is the first report to evaluate the roles of long/short fimbriae as well as gingipains on *P. gingivalis *biofilm formation.

Interestingly, the distinct fimbria types functioned differently in regard to biofilm formation. Our findings agree with a recent report [17], which suggested that long fimbriae are required for initial attachment and organization of biofilms. In that study, it was also shown that short fimbriae promoted bacterial autoaggregation, whereas long fimbriae suppressed it. Other studies have shown that autoaggregation is attributable to long fimbriae on the cell surface [18,31,32], and deletion of short fimbriae enhances autoaggregation [18], more consistent with our present findings. However, it would appear that autoaggregation is context and assay dependent, and in any event not a good predictor of accumulation on abiotic surfaces.

Recently, it was reported that ClpXP, a proteolytic core and associated ATPase unit of the bacterial stress response system, negatively regulated the surface exposure of short fimbriae, and a ClpXP mutant showed elevated monospecies biofilm formation [33]. As we have shown here that the short fimbria mutant MPG67 developed greater biofilm accumulation than the wild type, it is likely that ClpXP has numerous effects on cell surface molecules important in biofilm development.

The long/short fimbriae mutant MPG4167 and RgpA/B mutant KDP133 developed biofilms with significantly large amounts of bacterial cells. In addition, the exopolysaccharide/cell ratio was significantly smaller than the other strains, and the biofilms of these strains were shown to be fragile (Figures 5C and 6). Rgp is an enzyme that processes precursor proteins of bacterial surface components such as fimbriae [22,23], therefore, Rgp-null mutants exhibit defective surface protein presentation. Thus not only MPG4167 but also KDP133 do not have intact fimbrial protein on the cell surface, which might be related to imperfect anchoring of exopolysaccharide on the bacterial surfaces. The gingipains null mutant KDP136 did not show the same tendency in spite of the lack of both types of fimbriae, suggesting the presence of Kgp was related to the unusual exopolysaccharide accumulation. In contrast, long fimbriae mutant KDP150 formed a tough and cohesive biofilm, and its exopolysaccharide/cell ratio was significantly higher than the other strains. Together, these findings suggest that the exopolysaccharide/cell ratio seems to be related to the physical strength of *P. gingivalis *biofilms.

The specific role of Kgp may involve regulation of biofilm formation by the dispersion, de-concentration, and/or detachment of microcolonies. Rgp also seemed to coordinate the integrity of the biofilm in the developing phase as well as maturation phase. There are several reports which suggest that the present morphological changes in proteinase mutants were possibly due to loss of proteolytic activities. In *Staphylococcus aureus*, increased levels of serine proteases were detected in detaching biofilm effluents, and a serine protease inhibitor suppressed the biofilm detachment [34]. In the same report, a double mutant in a metalloprotease and serine proteases, which displayed minimal extracellular protease activity, showed significantly enhanced biofilm formation and a strongly attenuated detachment phenotype. In *Streptococcus pneumoniae*, trypsin or proteinase K was shown to inhibit biofilm development, and incubation of mature biofilms with proteinase K drastically diminished the number of biofilm-associated sessile cells [35]. Since our data also showed that the mutation in gingipain genes resulted in enhanced biofilm formation as well as a strongly attenuated detachment phenotype, this suggests that proteinase domains of Kgp and Rgp are significantly involved in biofilm regulation [5]. In addition, the tyrosine phosphatase Ltp1 reportedly dephosphorylated gingipains, resulting in suppression of biofilm formation [10], which also supports the involvement of gingipains as shown in this study. Furthermore, the present gingipain mutants lacked proteinase domains as well as C-terminal flanking segments coding for hemaglutinin/adhesin (HA) domains [36]. Higher concentrations of iron in the cultivation media can have a positive effect on the stability of the biofilms [37], thus decreased hemin uptake due to the lack of HA domains might modulate the biofilm structures in dTSB.

Autoaggregation driven by nonspecific hydrophobic mechanisms is thought to contribute to hetero- and homo-typic biofilm formation [24]. Indeed, the significant change of autoaggregation efficiencies in KDP129, KDP150, MPG67 and MPG4167 were found to be positively associated with alteration of biofilm structures under the non-proliferation condition. However, such an association was not observed in Rgp-null mutant strains, KDP133 and KDP136, and was not significant under the proliferation condition. Our present results suggested that a biofilm-regulatory molecule Rgp does not function through autoaggregation but rather through other mechanisms mediating intimate contact among *P. gingivalis *cells. Recently Kato *et al*. found that autoaggregation ability correlated poorly with the hydrophobicity in FimA-substituted mutants [38]. In addition, the hydrophobicity was reported not to depend on the presence or absence of FimA on the bacterial surface [39]. In-depth mathematical and physical examinations may be needed to explain the complicated roles of hydrophobicity, autoaggregation and cell surface structure on biofilm development.

Besides fimbria and proteinases, our findings indicate that other molecules of *P. gingivalis*, which are not processed by gingipains, mediate homotypic biofilm formation. Indeed several factors, including a putative glycosyltransferase (PG_0106), UDP-galactose 4-epimerase (GalE), internalin J protein (InlJ), a universal stress protein (UpsA), and a low molecular weight tyrosine phosphatase (Ltp1), have been reported to be required for homotypic biofilm formation by *P. gingivalis *[10,19,40-42]. Autoinducer-2, which regulates proteinase and hemagglutinin activities, hemin and iron acquisition pathways, and stress gene expression, is also considered to be involved in homotypic biofilm formation [43-46]. It is possible that these molecules also have effects in regard to biofilm structure alterations, in addition to fimbriae and gingipains. Further work is necessary to understand the complete process of the biofilm formation by *P. gingivalis*.

## Conclusion

The present results suggest distinct roles of long/short fimbriae and gingipains in homotypic biofilm development by *P. gingivalis*. Long fimbriae are initial positive mediators of biofilm formation, and thereafter they decrease the expression of exopolysaccharide to regulate adhesive properties. Short fimbriae as well as Kgp are negative regulators of microcolony formation. Rgp plays a bifunctional role to coordinate the integrity of the biofilm through mediating microcolony formation and restraining biovolume. Collectively, these molecules seem to act coordinately to regulate the development of mature biofilms.

## Methods

### Bacterial strains and media

The *P. gingivalis *strains used in this study are shown in Table 4. *P. gingivalis *cells were inoculated from blood agar plates and grown anaerobically (85% N_2_, 10% H_2_, 5% CO_2_) at 37°C in trypticase soy broth supplemented with 1 mg/ml of yeast extract, 1 μg/ml of menadione and 5 μg/ml of hemin (TSB). At stationary phase, the cells were harvested by centrifugation at 6,000 × *g *for 7 minutes, resuspended in pre-reduced 10 mM phosphate buffer containing 0.15 M sodium chloride (PBS; pH 7.4) and then used in the assays. When necessary, the following antibiotics were used at the concentrations shown in parentheses: chloramphenicol (20 μg/ml), erythromycin (10 μg/ml), and tetracycline (1 μg/ml). To observe initial attachment and organization of biofilms, *P. gingivalis *cells were anaerobically incubated in pre-reduced PBS without a nutrition source [19]. In order to monitor an increase in biovolume due to cell division as well as exopolysaccharide accumulation, bacterial cells were cultured in TSB medium diluted with PBS (dTSB; TSB/PBS ratio, 1:2) [47].

**Table 4:** *P. gingivalis *strains used in this study

Strain	Genotype	Relevant properties	Reference
33277	Wild type	Wild type	ATCC
KDP150	*fimA::erm*	Long fimbria (FimA)- deficient	[20]
MPG67	*mfa1::erm*	Short fimbria (Mfa1)- deficient	[18]
MPG4167	*fimA::erm mfa1::tetQ*	Long and short fimbria-deficient	[18]
KDP129	*kgp::cat*	Kgp-null	[20]
KDP133	*rgpA::tetQ rgpB::erm*	Rgp-null	[20]
KDP136	*rgpA::erm rgpB::tetQ kgp::cat*	Rgp/Kgp-null	[20]

### Autoaggregation assay

An autoaggregation assay was essentially performed as described previously [48]. Briefly, 1 ml of *P. gingivalis *suspension (4 × 10^8 ^cells) was transferred into a UV-cuvette then incubated at 37°C with stirring. Autoaggregation was monitored by measuring the decrease in optical density at *A*_550 _(OD_550_) using a UV-visible recording spectrophotometer (UV-265FW; Shimadzu Co. Kyoto, Japan). During the incubation, dA/dt was continuously calculated and recorded by subtraction of At, the absorbance at time t min, from At+, at time (t + 1) min. The maximum value of – dA/dt in this curve was used as the autoaggregation activity [48]. The data represent the mean ± standard error of three separate experiments with each strain in duplicate.

### Saliva

Saliva stimulated by mastication of paraffin balls was collected in a sterile centrifuge tube on ice from healthy donors and pooled, as described previously [49]. Dithiothreitol (Sigma-Aldrich, St. Louis, MO) was added to a 2.5 mM final concentration, then the saliva was gently stirred on ice for 10 minutes and centrifuged at 3,000 × *g *for 20 minutes at 4°C. The clarified saliva supernatant was decanted, 3 volumes of distilled water was added, and the 25% saliva was filtered through a 0.20 μm pore size filter and frozen in 40 ml aliquots. Immediately prior to use, the sterile saliva was thawed at 37°C; the slight precipitate was pelleted at 1,430 × *g *for 5 min, and the clear 25% saliva supernatant was used in experiments.

### Microscope observation

Quantitative and structural analysis of homotypic *P. gingivalis *biofilms was accomplished by confocal laser scanning microscopy (CLSM, Radiance 2100, Bio-Rad) and subsequent image analysis [50]. *P. gingivalis *was stained with CFSE (8 μg/ml; Molecular Probes, Eugene, OR), washed three times and 1 × 10^8 ^cells in PBS or dTSB were anaerobically incubated in a 25% saliva-coated wells of a chambered coverglass system (Culture Well™, Grace Bio Labs, Bend, OR) for 24 hours at 37°C in the dark on a rotator. The resulting biofilms were examined using the CLSM with reflected laser light at 488 nm. The images were analyzed using the Image J 1.34s (National Institutes of Health; Bethesda, MD) and Imaris 5.0.1 (Bitplane AG; Zurich, Switzerland) software packages. The experiment was repeated independently three times with each strain in triplicate.

### Biofilm characterization by image analysis

*Z *stacks of the *x-y *sections in the CLSM images were converted to composite images with the "Iso Surface" function of the "Surpass" option provided by Imaris 5.0.1 (Bitplane AG; Zurich, Switzerland) software. Iso Surface images were created at a threshold of 40 and smoothed with the "Gaussian Filter" function at a width of 1.28 μm, then the biovolume was calculated. Measurement of peak parameters was performed as described previously [50]. Digitally reconstructed images of the *x-z *section, 189.4 μm × appropriate height with 10-μm spaced *y*-series slices, were created using the "Reslice" function of Image J. An image series of the *x-z *section was processed using the "Find Edges" function, then the peak height was calculated by Image J. Color images of the *x-z *section were converted into gray scale and the density per vertical position (*x*-axis) was analyzed with the "Plot profile" function of Image J. The data were then exported as plot values with *x*-axis distance information. Peaks were defined as positions where plot values were higher than on either side, and the distance between two peaks was measured. The peak number was counted in a 90-μm section of the *x*-axis.

### Exopolysaccharide production assay

*P. gingivalis *organisms were stained with DAPI (50 μg/ml; Molecular Probes, Eugene, OR), then washed and cultured in 25% saliva-coated wells of CultureWell chambered coverglass system with dTSB for 24 hours. The resulting biofilms were washed, then exopolysaccharide was labelled with Concanavalin A-FITC and Wheat germ agglutinin-FITC (100 μg/ml; Molecular Probes) for 30 minutes at room temperature, as described previously [10]. After washing, fluorescent images were obtained using CLSM with reflected laser light at 405 and 488 nm, then analyzed as described above. The images were obtained with 8 fields per a sample. The experiment was repeated independently three times.

### Sonic disruption assay

A 12-well polystyrene plate (#1820-024, AGC Techno Glass, Chiba, Japan) was coated with 25% saliva. *P. gingivalis *cells (4 × 10^8 ^cfu/well) were incubated in a static manner in dTSB for 60 hours at 37°C and the resulting biofilms were sonicated for 1 second at output level 1 (output power: 25 W, oscillating frequency: 28 kHz, tip diameter: 2.5 mm) with a Handy ultrasonic disruptor (UR-20P, Tomy Seiko, Tokyo, Japan). During sonication, the oscillator was fixed with a stand, and the tip of horn was positioned 5 mm above from the center point of flat well bottoms. Immediately after the sonication, supernatants containing floating cells were removed by aspiration and the remaining biofilms were gently washed with PBS. *P. gingivalis *genomic DNA was isolated from the biofilms and the number of *P. gingivalis *cells per well was determined using real-time PCR, as described previously [51]. The data represent the means ± standard error of three separate experiments with each strain in duplicate.

### Statistical analyses

All data are expressed as the mean ± standard error. Multiple comparisons were performed by one-way analysis of variance and Sheffe's test using the SPSS 16.0J software (SPSS Japan Inc., Tokyo).

## Abbreviations

TSB: trypticase soy broth supplemented with 1 mg/ml of yeast extract, 1 μg/ml of menadione and 5 μg/ml of hemin; DAPI: 4',6-diamino-2-phenylindole; FITC: fluorescein isothiocyanate; CFSE: 5-(and-6)-carboxyfluorescein succinimidyl ester; CLSM: confocal laser scanning microscopy; dTSB: diluted TSB medium.

## Authors' contributions

MK carried out the microscope observation, image analysis and autoaggregation assay, as well as prepared the initial draft of the manuscript. AA conceived of the study and helped to draft the manuscript. EH and YY carried out the sonic disruption assay. HI performed the statistical analysis. KN and NH provided *P. gingivalis *knockout mutants used in this study. GDT developed the exopolysaccharide assay for *P. gingivlais*. RJL participated in the design of the study and helped to draft the manuscript. SS participated in the design of the study and coordination. All authors read and approved the final manuscript.

## References

[B1] Lamont RJ Jenkinson HF Life below the gum line: pathogenic mechanisms of *Porphyromonas gingivalis* Microbiol Mol Biol Rev 1998 62 1244 1263 9841671 10.1128/mmbr.62.4.1244-1263.1998 PMC98945

[B2] Holt SC Ebersole JL *Porphyromonas gingivalis*, *Treponema denticola*, and *Tannerella forsythia*: the "red complex", a prototype polybacterial pathogenic consortium in periodontitis Periodontol 2000 2005 38 72 122 10.1111/j.1600-0757.2005.00113.x 15853938

[B3] Imamura T The role of gingipains in the pathogenesis of periodontal disease J Periodontol 2003 74 111 118 10.1902/jop.2003.74.1.111 12593605

[B4] Paramonov N Rangarajan M Hashim A Gallagher A Aduse-Opoku J Slaney JM Hounsell E Curtis MA Structural analysis of a novel anionic polysaccharide from *Porphyromonas gingivalis *strain W50 related to Arg-gingipain glycans Mol Microbiol 2005 58 847 863 10.1111/j.1365-2958.2005.04871.x 16238632

[B5] Kadowaki T Nakayama K Okamoto K Abe N Baba A Shi Y Ratnayake DB Yamamoto K *Porphyromonas gingivalis *proteinases as virulence determinants in progression of periodontal diseases J Biochem 2000 128 153 159 10920248 10.1093/oxfordjournals.jbchem.a022735

[B6] Chen T Duncan MJ Gingipain adhesin domains mediate *Porphyromonas gingivalis *adherence to epithelial cells Microb Pathog 2004 36 205 209 10.1016/j.micpath.2003.12.001 15001226

[B7] Labrecque J Bodet C Chandad F Grenier D Effects of a high-molecular-weight cranberry fraction on growth, biofilm formation and adherence of *Porphyromonas gingivalis* J Antimicrob Chemother 2006 58 439 443 10.1093/jac/dkl220 16735419

[B8] Yamanaka A Kouchi T Kasai K Kato T Ishihara K Okuda K Inhibitory effect of cranberry polyphenol on biofilm formation and cysteine proteases of *Porphyromonas gingivalis* J Periodontal Res 2007 42 589 592 10.1111/j.1600-0765.2007.00982.x 17956474

[B9] Yamada M Ikegami A Kuramitsu HK Synergistic biofilm formation by *Treponema denticola *and *Porphyromonas gingivalis* FEMS Microbiol Lett 2005 250 271 277 16085371 10.1016/j.femsle.2005.07.019

[B10] Maeda K Tribble GD Tucker CM Anaya C Shizukuishi S Lewis JP Demuth DR Lamont RJ A *Porphyromonas gingivalis *tyrosine phosphatase is a multifunctional regulator of virulence attributes Mol Microbiol 2008 69 1153 1164 10.1111/j.1365-2958.2008.06338.x 18573179 PMC2537464

[B11] Amano A Nakagawa I Okahashi N Hamada N Variations of *Porphyromonas gingivalis *fimbriae in relation to microbial pathogenesis J Periodontal Res 2004 39 136 142 10.1111/j.1600-0765.2004.00719.x 15009522

[B12] Hajishengallis G Harokopakis E *Porphyromonas gingivalis *interactions with complement receptor 3 (CR3): innate immunity or immune evasion? Front Biosci 2007 12 4547 4557 10.2741/2409 17485396

[B13] Hajishengallis G Wang M Liang S Triantafilou M Triantafilou K Pathogen induction of CXCR4/TLR2 cross-talk impairs host defense function Proc Natl Acad Sci USA 2008 105 13532 13537 18765807 10.1073/pnas.0803852105 PMC2533224

[B14] Amano A Disruption of epithelial barrier and impairment of cellular function by *Porphyromonas gingivalis* Front Biosci 2007 12 3965 3974 10.2741/2363 17485350

[B15] Kuboniwa M Hasegawa Y Mao S Shizukuishi S Amano A Lamont RJ Yilmaz O *P. gingivalis *accelerates gingival epithelial cell progression through the cell cycle Microbes Infect 2008 10 122 128 18280195 10.1016/j.micinf.2007.10.011 PMC2311419

[B16] Park Y Simionato MR Sekiya K Murakami Y James D Chen W Hackett M Yoshimura F Demuth DR Lamont RJ Short fimbriae of *Porphyromonas gingivalis *and their role in coadhesion with *Streptococcus gordonii* Infect Immun 2005 73 3983 3989 15972485 10.1128/IAI.73.7.3983-3989.2005 PMC1168573

[B17] Lin X Wu J Xie H *Porphyromonas gingivalis *minor fimbriae are required for cell-cell interactions Infect Immun 2006 74 6011 6015 16988281 10.1128/IAI.00797-06 PMC1594877

[B18] Umemoto T Hamada N Characterization of biologically active cell surface components of a periodontal pathogen. The roles of major and minor fimbriae of *Porphyromonas gingivalis* J Periodontol 2003 74 119 122 10.1902/jop.2003.74.1.119 12593606

[B19] Capestany CA Kuboniwa M Jung IY Park Y Tribble GD Lamont RJ Role of the *Porphyromonas gingivalis *InlJ protein in homotypic and heterotypic biofilm development Infect Immun 2006 74 3002 3005 16622239 10.1128/IAI.74.5.3002-3005.2006 PMC1459709

[B20] Shi Y Ratnayake DB Okamoto K Abe N Yamamoto K Nakayama K Genetic analyses of proteolysis, hemoglobin binding, and hemagglutination of *Porphyromonas gingivalis*. Construction of mutants with a combination of *rgpA, rgpB, kgp*, and *hagA* J Biol Chem 1999 274 17955 17960 10.1074/jbc.274.25.17955 10364243

[B21] Kadowaki T Nakayama K Yoshimura F Okamoto K Abe N Yamamoto K Arg-gingipain acts as a major processing enzyme for various cell surface proteins in *Porphyromonas gingivalis* J Biol Chem 1998 273 29072 29076 10.1074/jbc.273.44.29072 9786913

[B22] Nakayama K Yoshimura F Kadowaki T Yamamoto K Involvement of arginine-specific cysteine proteinase (Arg-gingipain) in fimbriation of *Porphyromonas gingivalis* J Bacteriol 1996 178 2818 2824 8631669 10.1128/jb.178.10.2818-2824.1996 PMC178016

[B23] Shoji M Naito M Yukitake H Sato K Sakai E Ohara N Nakayama K The major structural components of two cell surface filaments of *Porphyromonas gingivalis *are matured through lipoprotein precursors Mol Microbiol 2004 52 1513 1525 10.1111/j.1365-2958.2004.04105.x 15165251

[B24] Kolenbrander PE Palmer RJ Jr Rickard AH Jakubovics NS Chalmers NI Diaz PI Bacterial interactions and successions during plaque development Periodontol 2000 2006 42 47 79 10.1111/j.1600-0757.2006.00187.x 16930306

[B25] Kato T Tsuda T Omori H Kato T Yoshimori T Amano A Maturation of fimbria precursor protein by exogenous gingipains in *Porphyromonas gingivalis *gingipain-null mutant FEMS Microbiol Lett 2007 273 96 102 10.1111/j.1574-6968.2007.00779.x 17559394

[B26] Jenkinson HF Lamont RJ Oral microbial communities in sickness and in health Trends Microbiol 2005 13 589 595 10.1016/j.tim.2005.09.006 16214341

[B27] Kuramitsu HK He X Lux R Anderson MH Shi W Interspecies interactions within oral microbial communities Microbiol Mol Biol Rev 2007 71 653 670 18063722 10.1128/MMBR.00024-07 PMC2168648

[B28] Lamont RJ Jenkinson HF Subgingival colonization by *Porphyromonas gingivalis* Oral Microbiol Immunol 2000 15 341 349 10.1034/j.1399-302x.2000.150601.x 11154429

[B29] O'Toole GA Microbiology: Jekyll or hide? Nature 2004 432 680 681 10.1038/432680a 15592392

[B30] Stoodley P Sauer K Davies DG Costerton JW Biofilms as complex differentiated communities Annu Rev Microbiol 2002 56 187 209 10.1146/annurev.micro.56.012302.160705 12142477

[B31] Andrian E Grenier D Rouabhia M *Porphyromonas gingivalis*-epithelial cell interactions in periodontitis J Dent Res 2006 85 392 403 10.1177/154405910608500502 16632751

[B32] Kuramitsu H Tokuda M Yoneda M Duncan M Cho MI Multiple colonization defects in a cysteine protease mutant of *Porphyromonas gingivalis* J Periodontal Res 1997 32 140 142 10.1111/j.1600-0765.1997.tb01395.x 9085224

[B33] Capestany CA Tribble GD Maeda K Demuth DR Lamont RJ Role of the Clp system in stress tolerance, biofilm formation, and intracellular invasion in *Porphyromonas gingivalis* J Bacteriol 2008 190 1436 1446 18065546 10.1128/JB.01632-07 PMC2238200

[B34] Boles BR Horswill AR Agr-mediated dispersal of *Staphylococcus aureus *biofilms PLoS Pathog 2008 4 e1000052 18437240 10.1371/journal.ppat.1000052 PMC2329812

[B35] Moscoso M Garcia E Lopez R Biofilm formation by *Streptococcus pneumoniae*: role of choline, extracellular DNA, and capsular polysaccharide in microbial accretion J Bacteriol 2006 188 7785 7795 16936041 10.1128/JB.00673-06 PMC1636320

[B36] Potempa J Mikolajczyk-Pawlinska J Brassell D Nelson D Thogersen IB Enghild JJ Travis J Comparative properties of two cysteine proteinases (gingipains R), the products of two related but individual genes of *Porphyromonas gingivalis* J Biol Chem 1998 273 21648 21657 10.1074/jbc.273.34.21648 9705298

[B37] Mohle RB Langemann T Haesner M Augustin W Scholl S Neu TR Hempel DC Horn H Structure and shear strength of microbial biofilms as determined with confocal laser scanning microscopy and fluid dynamic gauging using a novel rotating disc biofilm reactor Biotechnol Bioeng 2007 98 747 755 10.1002/bit.21448 17421046

[B38] Kato T Kawai S Nakano K Inaba H Kuboniwa M Nakagawa I Tsuda K Omori H Ooshima T Yoshimori T Amano A Virulence of *Porphyromonas gingivalis *is altered by substitution of fimbria gene with different genotype Cell Microbiol 2007 9 753 765 10.1111/j.1462-5822.2006.00825.x 17081195

[B39] Hamada N Watanabe K Sasakawa C Yoshikawa M Yoshimura F Umemoto T Construction and characterization of a fimA mutant of *Porphyromonas gingivalis* Infect Immun 1994 62 1696 1704 7909537 10.1128/iai.62.5.1696-1704.1994 PMC186386

[B40] Davey ME Duncan MJ Enhanced biofilm formation and loss of capsule synthesis: deletion of a putative glycosyltransferase in *Porphyromonas gingivalis* J Bacteriol 2006 188 5510 5523 16855241 10.1128/JB.01685-05 PMC1540017

[B41] Nakao R Senpuku H Watanabe H *Porphyromonas gingivalis galE *is involved in lipopolysaccharide O-antigen synthesis and biofilm formation Infect Immun 2006 74 6145 6153 16954395 10.1128/IAI.00261-06 PMC1695533

[B42] Chen W Honma K Sharma A Kuramitsu HK A universal stress protein of *Porphyromonas gingivalis *is involved in stress responses and biofilm formation FEMS Microbiol Lett 2006 264 15 21 10.1111/j.1574-6968.2006.00426.x 17020544

[B43] Burgess NA Kirke DF Williams P Winzer K Hardie KR Meyers NL Aduse-Opoku J Curtis MA Camara M LuxS-dependent quorum sensing in *Porphyromonas gingivalis *modulates protease and haemagglutinin activities but is not essential for virulence Microbiology 2002 148 763 772 11882711 10.1099/00221287-148-3-763

[B44] Chung WO Park Y Lamont RJ McNab R Barbieri B Demuth DR Signaling system in *Porphyromonas gingivalis *based on a LuxS protein J Bacteriol 2001 183 3903 3909 11395453 10.1128/JB.183.13.3903-3909.2001 PMC95272

[B45] James CE Hasegawa Y Park Y Yeung V Tribble GD Kuboniwa M Demuth DR Lamont RJ LuxS involvement in the regulation of genes coding for hemin and iron acquisition systems in *Porphyromonas gingivalis* Infect Immun 2006 74 3834 3844 16790755 10.1128/IAI.01768-05 PMC1489751

[B46] Yuan L Hillman JD Progulske-Fox A Microarray analysis of quorum-sensing-regulated genes in *Porphyromonas gingivalis* Infect Immun 2005 73 4146 4154 15972504 10.1128/IAI.73.7.4146-4154.2005 PMC1168601

[B47] Chen W Palmer RJ Kuramitsu HK Role of polyphosphate kinase in biofilm formation by *Porphyromonas gingivalis* Infect Immun 2002 70 4708 4715 12117989 10.1128/IAI.70.8.4708-4715.2002 PMC128176

[B48] Nagata H Murakami Y Inoshita E Shizukuishi S Tsunemitsu A Inhibitory effect of human plasma and saliva on co-aggregation between *Bacteroides gingivalis *and *Streptococcus mitis* J Dent Res 1990 69 1476 1479 2384623 10.1177/00220345900690080501

[B49] Palmer RJ Jr Kazmerzak K Hansen MC Kolenbrander PE Mutualism versus independence: strategies of mixed-species oral biofilms in vitro using saliva as the sole nutrient source Infect Immun 2001 69 5794 5804 11500457 10.1128/IAI.69.9.5794-5804.2001 PMC98697

[B50] Kuboniwa M Tribble GD James CE Kilic AO Tao L Herzberg MC Shizukuishi S Lamont RJ *Streptococcus gordonii *utilizes several distinct gene functions to recruit *Porphyromonas gingivalis *into a mixed community Mol Microbiol 2006 60 121 139 10.1111/j.1365-2958.2006.05099.x 16556225

[B51] Kuboniwa M Amano A Kimura KR Sekine S Kato S Yamamoto Y Okahashi N Iida T Shizukuishi S Quantitative detection of periodontal pathogens using real-time polymerase chain reaction with TaqMan probes Oral Microbiol Immunol 2004 19 168 176 10.1111/j.0902-0055.2004.00135.x 15107068

